# Persistent Increased Frequency of Genomic Instability in Women Diagnosed with Breast Cancer: Before, during, and after Treatments

**DOI:** 10.1155/2018/2846819

**Published:** 2018-06-14

**Authors:** Márcia Fernanda Correia Jardim Paz, André Luiz Pinho Sobral, Jaqueline Nascimento Picada, Ivana Grivicich, Antonio Luiz Gomes Júnior, Ana Maria Oliveira Ferreira da Mata, Marcus Vinícius Oliveira Barros de Alencar, Rodrigo Mendes de Carvalho, Kátia da Conceição Machado, Muhammad Torequl Islam, Ana Amélia de Carvalho Melo Cavalcante, Juliana da Silva

**Affiliations:** ^1^Laboratory of Genetic Toxicology, PPGBioSaúde and PPGGTA, Lutheran University of Brazil (ULBRA), Av. Farroupilha 8001, Prédio 22, Sala 22 (4° Andar), 92425-900 Canoas, RS, Brazil; ^2^Laboratory of Genetic Toxicology, PPGCF, Federal University of Piauí, Av. Universitária S/N, Ininga, 64049-550 Teresina, PI, Brazil; ^3^Post-Graduation Program in Biotechnology, RENORBIO, Federal University of Piauí, Av. Universitária, S/N, Ininga, 64049-550 Teresina, PI, Brazil; ^4^University Hospital of Piauí, Av. Universitária, S/N, Ininga, 64049-550 Teresina, PI, Brazil; ^5^Laboratory of Cancer Biology, PPGBioSaúde and PPGGTA, Lutheran University of Brazil (ULBRA), Av. Farroupilha 8001, Prédio 22, Sala 22 (4° Andar), 92425-900 Canoas, RS, Brazil; ^6^Biomedicine Department, UNINOVAFAPI University, Teresina, Brazil; ^7^Department of Biochemistry and Pharmacology, Federal University of Piauí, Av. Universitária, S/N, Ininga, 64049-550 Teresina, PI, Brazil; ^8^Central Laboratory of Public Health of Piauí, Rua Dezenove de Novembro 1945, Bairro Primavera, 64002-570 Teresina, PI, Brazil; ^9^Department for Management of Science and Technology Development, Ton Duc Thang University, Ho Chi Minh City 700000, Vietnam; ^10^Faculty of Pharmacy, Ton Duc Thang University, Ho Chi Minh City 700000, Vietnam

## Abstract

This study aimed to evaluate DNA damage in patients with breast cancer before treatment (background) and after chemotherapy (QT) and radiotherapy (RT) treatment using the Comet assay in peripheral blood and the micronucleus test in buccal cells. We also evaluated repair of DNA damage after the end of RT, as well as the response of patient's cells before treatment with an oxidizing agent (H_2_O_2_; challenge assay). Fifty women with a mammographic diagnosis negative for cancer (control group) and 100 women with a diagnosis of breast cancer (followed up during the treatment) were involved in this study. The significant DNA damage was observed by increasing in the index and frequency of damage along with the increasing of the frequency of micronuclei in peripheral blood and cells of the buccal mucosa, respectively. Despite the variability of the responses of breast cancer patients, the individuals presented lesions on the DNA, detected by the Comet assay and micronucleus Test, from the diagnosis until the end of the oncological treatment and were more susceptible to oxidative stress. We can conclude that the damages were due to clastogenic and/or aneugenic effects related to the neoplasia itself and that they increased, especially after RT.

## 1. Introduction

Breast cancer is a heterogeneous group of neoplasms originating from the epithelial cells lining the milk ducts and is a complex disease characterized by disordered cell growth involving different mechanisms [1]. Among cancers, breast cancer is the most common and lethal in women [2], with more than one million cases diagnosed worldwide annually [3–5]. In Brazil, it is the second most frequent cause of mortality among women; first is skin cancer [5, 6]. Breast cancer is a multifactorial disease, where epidemiological studies indicate that in addition to genetic predisposition, exposure to mutagenic agents, nutritional habits, and lifestyle are relevant factors that can trigger the carcinogenic process [7–9]. Associated with this, reproductive age, involving events such as menarche, menopause, pregnancy, and hormone therapy, also constitutes risks to induce neoplastic transformations [10, 11].

Early diagnosis indicates a good prognosis and is fundamental in patient survival, being able to signal a less aggressive treatment [12, 13]. Mammography remains the primary method of diagnosing breast cancer [14]. The performance of surgery, chemotherapy (QT), radiotherapy (RT), and, in some cases, hormone therapy is alternative that science has for the treatment of this pathology [15]. In recent years, the description of well-defined molecular subtypes of breast cancer, together with the identification of the driving genetic alterations and signaling pathways, has led to the clinical development of a number of successful molecular-targeted agents [4].

Cancer is intimately related to the accumulation of DNA damage, as well as with DNA repair failures. Cytogenetic biomarkers have attracted more attention from the scientific community because they are potential indicators of biological effects, including cancer risks [4, 16]. The use of Comet assay has been used to detect genotoxicity and to human biomonitoring [17]. In addition, the micronucleus test, which observes numerical chromosomal abnormalities (e.g., whole chromosomal lagging or malsegregation at mitosis) or from structural chromosomal abnormalities (e.g., the failure of an acentric fragment or dicentric chromosome to segregate at mitosis) or cell death, has been also used [18]. Besides, to assist in the diagnosis, these methodologies could be used to follow the patient in understanding their individual response to treatment choices.

Thus, the aim of this study was to evaluate DNA damage in patients with breast cancer before treatment (background) and after QT and RT treatment using the Comet assay in peripheral blood and micronucleus test in buccal cells. In addition, we evaluated recovery DNA damage after stop RT, as well as response of the patient's cells before treatment with an oxidizing agent (H_2_O_2_; challenge assay).

## 2. Materials and Methods

### 2.1. Ethics Statement

Human subject research was approved by the Centro Universitário UNINOVAFAPI (CONEP protocol number 0408.0.043.000-11). Written documentation of informed consent was obtained from all research participants.

### 2.2. Study Group and Sampling

A total of 100 patients presented with a diagnosis of breast cancer from the oncology clinic of the Hospital São Marcos (Piauí, Brazil) (followed up during the treatment; mean age 50.0 ± 12.0 years) and 50 women with a mammographic diagnosis negative for cancer (control group; mean age 47.0 ± 13.0 years). Patients with organic, renal, and hepatic dysfunction or other associated chronic disease were considered as exclusion criteria. Only 10% of patients was considered smokers. All volunteers answered an individual health questionnaire as recommended by the International Commission for Protection against Environmental Mutagens and Carcinogens [19].

The clinical stages of the patients associated with the histopathological results lead to the choice of the chemotherapeutic scheme. The patients in this study underwent two different QT schemes: (a) FAC, which represent 500 mg/m^2^ of 5-fluorouracil, 50 mg/m^2^ of doxorubicin, plus 500 mg/m^2^ of cyclophosphamide, in 21-day cycles; (b) AC, which represents 60 mg/m^2^ doxorubicin and 600 mg/m^2^ cyclophosphamide, also in 21-day cycles. Patients undergoing the AC regimen still receive 80 mg/m^2^ of taxol per week for 12 weeks, seeking a potentiation of this treatment. Regarding RT, patients were exposed to 25 adjuvant radiotherapy sessions, alone or after QT, with radiation doses of 4500 to 5000 cGy total and with 180 to 200 cGy/fraction.

The blood and buccal cells sampling were performed on the same days. In this study, five collections were performed in patients with breast cancer: (1) at the time of diagnosis, prior to treatment; (2) 3 weeks after begin chemotherapy, after the different QT schemes; (3) prior to RT initiation; (4) in the third week after RT initiation; and (5) 21 days after the end of the RT sessions.

### 2.3. Alkaline Comet Assay

Samples were processed immediately after collection using heparin tubes. The method was performed according to Tice et al. [20], and the slides were stained with silver solution as described in Nadin et al. [21]. The results were expressed as damage index (DI) and damage frequency (DF). For the evaluation of DNA damage, 100 cells per subject were analyzed at 200x magnification under a light microscope, using blinded slides. Cells were assessed visually and received scores from 0 (no migration) to 4 (maximal migration) according to tail intensity (size and shape). Therefore, the total scores (DI, arbitrary units) for 100 cells ranged from 0 (all cells with no migration) to 400 (all cells with maximal migration) [22]. Dusinska and Collins [23] demonstrated that results expressed as either % tail DNA or arbitrary units correlate extremely well. DF was calculated by subtracting 100 cells with zero damage, that is, based on the number of cells with damage versus those without damage. For assessment of susceptibility to exogenous DNA damage, two slides prepared from patients from diagnostic moment (before treatment) were exposed to 0.25 mM recently prepared H_2_O_2_ (challenge treatment) for 5 min, at 4°C [24]. After that, the slides were put in lysis solution for 1 h at 4°C. Subsequent steps were the same as in the alkaline version of the Comet assay.

### 2.4. Buccal Micronucleus Cytome Assay (BMNCyt)

The BMNCyt test in exfoliated epithelial cells of oral mucosa was performed according to the method described by Thomas et al. [25], with some alterations. Briefly, buccal cell samples were collected from the inner cheeks of the subjects with a cytobrush, which was immersed in 5 mL of cold saline solution (NaCl 0.9%), and after washed three times with saline the cells were fixated on Carnoy's solution. After the slides had been prepared, they were stained with Schiff's reagent and Light Green. Cells were evaluated according Thomas et al. [25] at 1000x magnification under a light microscope, using blinded slides. The BMNCyt assay has been used to measure biomarkers of DNA damage (micronuclei and/or elimination of nuclear material by budding, BUDs), cytokinetic defects (binucleated cells), and cell death (condensed chromatin, and karyorrhectic, pyknotic, and karyolitic cells). For each volunteer, 2.000 buccal cells (1000 from each of the duplicate slides) were scored.

### 2.5. Statistical Analysis

The normality of the variables was evaluated by the Kolmogorov-Smirnov test, and Student's *t*-test or Mann Whitney *U* test was used to compare the characteristics of the study population and DNA damage in relation to characteristics of the study population. The statistical differences of damage observed for groups by the comet assay and BMNCyt assay were determined by ANOVA test. Values of *P* < 0.05 were considered statistically significant. All analyses were performed using the GraphPad PRISM statistical software (GraphPad Inc., San Diego, CA, USA).

## 3. Results

In clinical diagnosis, tumor types were classified as 83% with invasive ductal carcinoma, 6% with invasive lobular carcinoma, 3% intraductal carcinoma, 3% medullary carcinoma, and 3% phyllodes tumor, presenting staging of I to III.

Damage index and micronucleus frequency (mean ± SD) during diagnostic of breast cancer in relation to clinical characteristics of patients are presented in Table 1. Individuals with negative receptors for estrogen and progesterone presented higher levels of DNA damage, observed by Comet assay, than positive ones.

The genotoxicity data evaluated with the comet assay in peripheral blood are shown in Table 2. All patient group demonstrated a significant increase of DNA damage in relation to control but not in relation to different groups.

The micronucleus test in buccal cells also showed DNA damage evidenced by the significant increase of micronuclei, BUDs, and binucleated cells. Cell death was also increased in the groups in relation to the control group (Table 3). In addition, an increase in DNA damage and cell death during treatment can be observed in relation to the patients at the time of diagnosis.

**Figure 1:**
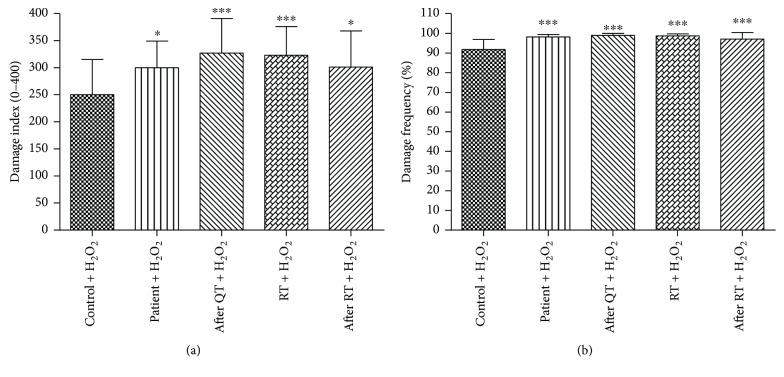
Damage index (a) and damage frequency (b) induced by H_2_O_2_ (challenge assay) to peripheral blood lymphocytes from breast cancer patients at diagnosis, during and after treatments and healthy controls. ^∗^Significance at *P* < 0.05 and ^∗∗∗^*P* < 0.001 compared to negative control (Kruskal-Wallis test).

Figure 1 shows that both DI and DF demonstrate a significant increase for challenge assay in all groups with breast cancer, from diagnosis to the end of radiotherapy treatment in relation to the control group exposed to H_2_O_2_.

**Figure 2:**
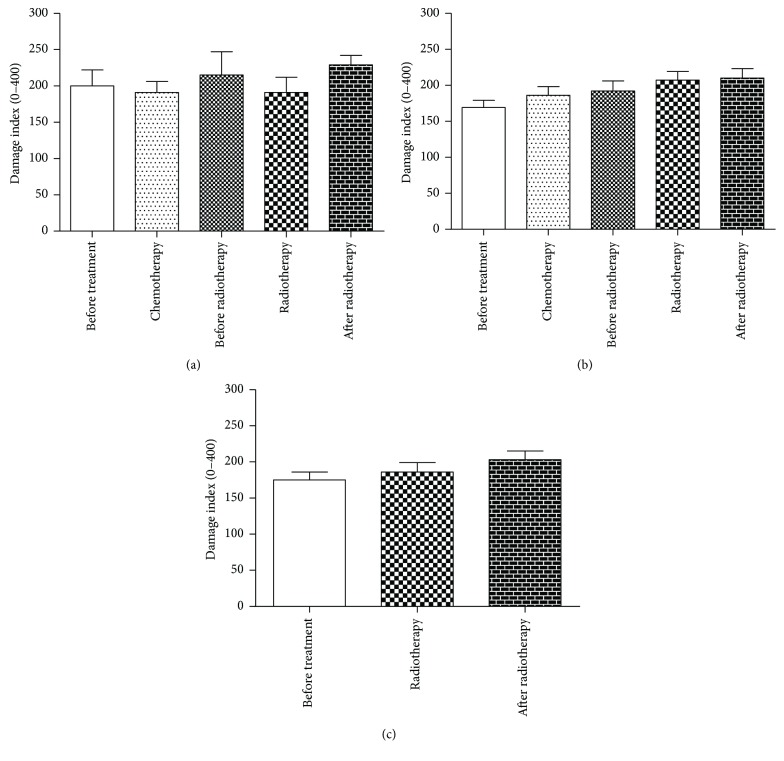
Damage index in relation to the therapeutic regimen: (a) FAC, (b) AC, and (c) RT-isolated.

**Figure 3:**
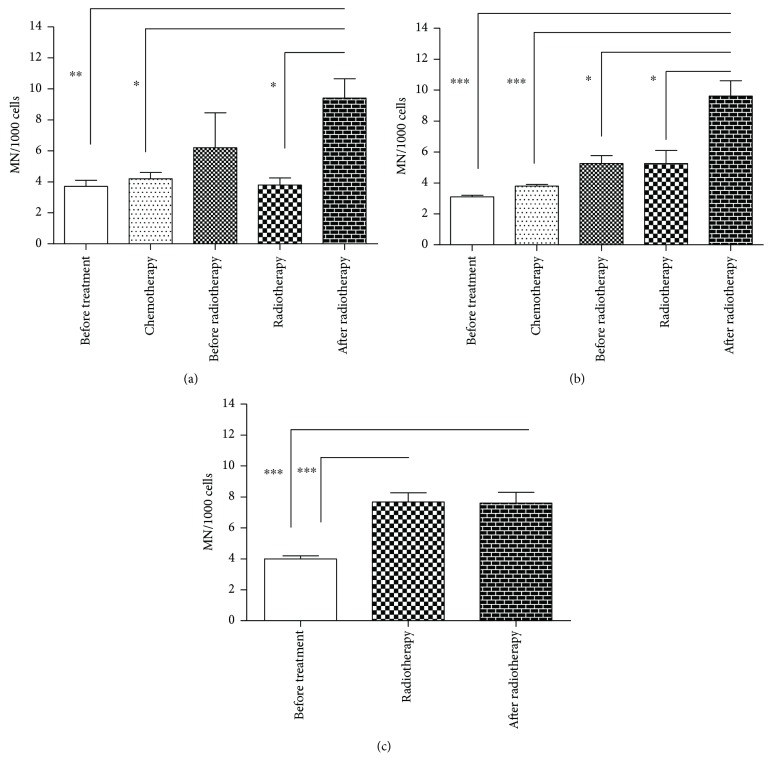
Micronucleus frequency in relation to the therapeutic regimen: (a) FAC, (b) AC, and (c) RT-isolated. ^∗^Significant at *P* < 0.05; ^∗∗^*P* < 0.01; ^∗∗∗^*P* < 0.001 using Kruskal-Wallis test.

Figures 2 and 3 demonstrate a relationship between DNA damage using comet assay and micronucleus test in relation to the different therapeutic regimens used by the patients in this study (AC, FAC, or RT-isolated). No difference was observed using comet assay, but micronucleus test for 21 days after the end of radiotherapy demonstrated the highest values of micronucleus for all therapeutic regimen.

## 4. Discussion

Breast cancer is one of the most relevant causes of death among women worldwide [3]. Data from the World Cancer Report of the International Agency for Research on Cancer (IARC) and the World Health Organization (WHO) show a 2030 incidence of 27 million cases, resulting in 17 million deaths and 75 million people annually, with cancer. This increasing occurrence [26] characterizes it as one of the most important public health problems today. This pathology, which, in the 70s, was the fourth leading cause of death, currently occupies the second position of the global incidence [27]. Understanding the risk factors for breast cancer is of paramount importance for epidemiological, social, and individual studies and is critical for the development of prevention strategies and therapies [28].

Another crucial factor for this pathology is late diagnosis, which signals advanced stages of the disease. Clinical stages 0, I, and II of the American Joint Committee on Cancer system, which considers the extent of primary tumor and metastases, are classified as an early stage of breast cancer; late-stage patients belong to the groups III and IV [29]. In our study, although 41% of the patients had stages III and IV, signaling an advanced disease, there was no statistically significant correlation between the DI and micronucleus (MN) frequency of the patients at diagnosis. This fact may be related to the sensitivity of the comet assay, as a marker of genomic instability, and could be used since the beginning of the disease. Genetic alterations, including telomere damage, chromosomal aberrations and amplification, and epigenetic modifications, are an initial step in the process of carcinogenesis [30] and tumor progression [31]. Thus, the genomic instability, detected by the “comet assay and MN test”, can be suggested as markers for cancer [32, 33], and its monitoring is important in therapeutics, especially with the changes in the chromosomes [31, 34].

Breast cancer is a heterogeneous disease with multiple types of intrinsic tumors [35], which can be classified into distinct subgroups presenting different biological, clinical, and behavioral parameters offered by immunohistochemical examination. This biomarker is important for oncology, since it has information of prognostic value and predictive response to certain therapies, both for metastatic disease and adjuvant therapy [36]. The literature reports that hormone receptor positivity confers a better prognosis to metastatic disease. Its major relevance lies in the signaling of specific therapies [37]. The biomarkers Her2, ER, PR, and Ki-67, associated with the clinical and histopathological stages, guide the therapeutic management of cancer patients. In our study, 67% and 72% of the patients presented positivity for estrogen and progesterone receptors, respectively. Although the negativity for ER and PR was lower, 33% and 28%, respectively, the patients at diagnosis showed DNA damage as evidenced by the statistically significant increase in DI and frequency of MN. Júnior et al. [38] corroborated with the data obtained in a study when monitoring patients with breast cancer who, at the time of diagnosis, already presented damages in the genetic materials (e.g., DNA and RNA), demonstrating genomic instability. The request for the immunohistochemical test for Ki-67 also has a prognostic and therapeutic decision impact on breast cancer because it is a marker of cell proliferation [39]. In our study, 78% of the patients presented high Ki-67, suggestive of a disease with a more aggressive biological behavior. However, in this study, no influence of this factor on genetic damage was observed.

Early diagnosis indicates a good prognosis and is fundamental to patient survival [40, 41]; that for nonmetastatic disease, the treatment options fall into surgery (radical mastectomy or conservative surgery), QT, RT, QT, and hormone therapy [42]. RT and QT, which have the cytotoxic capacity to kill cancer cells, are one of the pillars of oncology therapy used by half the cancer population [43]. The planning and association of QT, RT, and surgery have increased the survival of cancer patients. However, the radiosensitivity and radioresistance presented by ionizing radiation have contributed to the limitation of therapeutic success [44]. Similar to RT, chemotherapeutics also have limitations. DNA damage assessed by the comet assay in peripheral blood showed a statistically significant increase in all the treatment steps and protocols, as well as in the micronucleus test, when compared to the control group, especially for the group after radiotherapy (after 21 days). Similar to our results, other authors have observed increased DNA damage by the comet assay and micronucleus test in breast cancer patients in different treatments and protocols [38, 45].

Studies developed by Iarmacovai et al. [46], conducting a meta-analysis of the frequency of MN in peripheral blood lymphocytes of cancer patients, evidenced a significant increase in the frequency of this biomarker in patients not treated with antineoplastic therapy. Corroborating with these data, Santos et al. [47] demonstrated the high frequency of MN in peripheral blood lymphocytes in 45 women with untreated invasive or in situ breast cancer. Murgia et al. [48], analyzing peripheral blood lymphocytes of 1650 individuals without diseases, showed strong predictive values of MN frequency associated with the risk of cancer death. In this study, significant increases were observed in MN frequencies in all groups, from diagnosis (baseline damage) to after RT, in relation to the control group and to baseline damage. However, after RT, the data were significant compared to before radiotherapy (after chemotherapy), indicating that, after RT, the patients were more genetically unstable due to the probable aneugenic and/or clastogenic effects, considering this biomarker of mutagenicity. Other studies have also pointed to DNA damage in patients with breast cancer exposed to RT by a significant increase in breast cancer during cancer treatment [49]. MN are simple markers routinely examined in cytological preparations, ensuring credibility in the assessment of cytogenetic damage of populations exposed to mutagenic and carcinogenic agents [50, 51]. As they result from aggressions in the genetic material, they represent a potential risk for the onset of cancer [8, 52]. It has been reported that the frequency of MN resulting from exposure to IR is dose-dependent [53].

Bonassi et al. [8] have shown evidence that the frequency of MN in peripheral blood lymphocytes is predictive of cancer risk, suggesting that increased MN formation is associated with the latest events in carcinogenesis. Similar to our data, in a review of human biomonitoring study with application of the MN Test, Speit et al. [54] indicate that the therapies used in cancer patients, QT and RT, result in an increase in MN formation due to aggression to the genetic material. By the micronucleus test, it has been observed the increase of nuclear buds only after RT and binucleate cells in QT, RT, and after RT. It is known that the nuclear bud formation may be related to the chromosomal instabilities resulting from genetic material damage or to gene amplification [55, 56]. The presence of binucleate cells is related to cytokinesis failures and to the occurrence of aneuploidies resulting from the cytotoxic activity of chemotherapeutics [55–57]. Most chemotherapeutics, used in clinical practice, have diverse mechanism of actions that converge for changes in the cell cycle and consequent impairment of cell division and cell death. During this process, chemotherapeutic treatment can alter the final events of the cell division, leading to blocking of cytokinesis and formation of binucleate cells. Despite these findings, Torres-Bugarín et al. [58], studying genotoxic QT effects in 163 patients with various cancers, found a decrease in the frequency of binucleate cells throughout the treatment, justified by the fact that QT leads to cell death before the end of the cell cycle. In the present study, an increase of karyorrhectic cells due to QT and pyknotic due to RT was observed, and the pyknotic cells remained increased after RT compared to the pretreatment group, indicating increased cell death by both QT and RT.

Antineoplastic agents, classified as cytotoxic, include chemical agents that control the development of tumors by killing actively growing cells. Among these, doxorubicin, which despite its great therapeutic potential in a wide variety of cancers [59], is limited by the severe side effects such as a cardiotoxicity present in 50% of patients and myelosuppression. Exposure of the DNA molecule to radiation induces a signal transduction cascade resulting in damage to the genetic material, including the increase of reactive oxygen species (ROS) [60]. There are records that signal IRs as responsible for the induction of chromosomal aberrations (AC) and apoptosis [61]. Tumor suppressor genes, such as p53 and *PTEN*, can be dysregulated, resulting in impairment of important functions such as induction of apoptosis, activation of the repair system, and cell cycle arrest [62]. Thus, ROS, by different mechanisms of action, can lead to apoptosis and tumor regression. In this study, when the patients' samples were challenged to use an oxidizing agent (H_2_O_2_) in the different treatments, we observed a significant increase of damages when compared to the samples of the control subjects (without cancer) exposed to the agent, which shows a susceptibility of these individuals to agents inducing oxidative damage. Blasiak et al. [63] also reported sensitivity of lymphocytes from BC patients to hydrogen peroxide (H_2_O_2_). Despite this, the control group shows a DI (comet assay) of about 22.9, while patients shows a DI of 180.9 before any treatment. However, after treatment with an oxidizing agent, the average DI of the controls rises ~10 times and patients about ~1.7 times. Therefore, patients' cells appear relatively less susceptible to oxidative damage than cells from individuals without cancer. Possibly the dose of H_2_O_2_ was high, which probably saturated the detection capacity of damage for patients by comet assay (reaching a limit of damage, almost 100% of damage). Brandão et al. [64], in a study on H_2_O_2_-induced cytotoxicity in human cells deficient in DNA repair, revealed that deficient lines in the nucleotide excision repair pathway were more sensitive to an inducer of oxidative damage, such as H_2_O_2_. In view of the above, a deficiency in the repair system may justify the potentiation of the peroxide sensitivity, culminating in the increase of damages.

Due to the different effects that could be induced by the different chemotherapeutic treatments, a comparison between induction of an increase in the DI and MN and the different treatment protocols was performed. Our results demonstrate that although there was no statistically significant increase in DI, the frequency of MN was statistically significant in the different cancer protocols, FAC, AC, and RT-isolated. Guerreiro et al. [65] report an increased frequency of MN and binucleate in breast cancer cells exposed to DOX. These data corroborate with Uriol et al. [66] who report that most chemotherapeutic treatments induce different DNA damage as observed in this study.

The different DNA damage induced by antineoplastics by DNA is associated with the different classes of these agents. Chemotherapy drugs classified as antimetabolites include compounds of clinical use that have different mechanisms of action that interfere with the synthesis of new precursors of DNA and RNA, inhibitors of DNA synthesis and compounds that alter the pattern of DNA methylation. As an example, there is 5-fluorouracil, which is an antimetabolite analogous to pyrimidine. Although the mechanisms of action of anthracyclines, including doxorubicin, are still controversial, we can consider DNA intercalation, free radical generation, DNA alkylation, and covalent bonding between DNA strands (DNA crosslinks) [67]. Of the natural products, taxol is a drug that acts as a poison of the mitotic spindle, increasing the polymerization of tubulin. These antimitotic agents stimulate the polymerization of the microtubules. This site-specific binding seems to antagonize the breakdown of this cytoskeletal key protein, with consequent formation of stable and abnormal microtubules, blocking the progression of G2 and M phase in the cell cycle [68–70].

The comet assay is increasingly being used to detect genotoxicity and human biomonitoring [38, 71], as well as the MN test, which can detect clastogenesis, aneugenesis, and cell death [18, 52]. Biomonitoring of molecular alterations can be an important tool to better understand the molecular biology of cancer, resulting in accurate diagnoses and successful treatments, especially due to the lack of specificity and selectivity in cancer therapy [72]. To this end, cytogenetic biomarkers have attracted more attention from the scientific community because they are potential indicators of biological effects, including cancer risks.

## Figures and Tables

**Table 1:** Damage index and micronucleus frequency (mean ± SD) during diagnostic of breast cancer in relation to clinical characteristics of patients.

Parameters	Characteristics (*n*)	Damage index (0–400)	MN/1000 cells
Family breast cancer	No (58)	201.30 ± 59.21	4.09 ± 1.92
	Yes (42)	196.90 ± 60.60	3.80 ± 1.25
Clinical staging	I and II (59)	194.80 ± 62.36	4.36 ± 1.60
	III and IV (41)	205.10 ± 55.64	3.18 ± 0.98
Estrogen receptors	Negative (33)	227.80 ± 48.58^∗∗∗^	4.09 ± 1.22
	Positive (67)	185.00 ± 59.61	3.94 ± 1.66
Progesterone receptors	Negative (28)	218.30 ± 56.31^∗^	3.70 ± 1.15
	Positive (72)	192.10 ± 59.50	4.05 ± 1.66
Her-2	Negative (30)	183.80 ± 63.56	3.75 ± 1.39
	Positive (70)	203.60 ± 57.86	3.62 ± 1.40
Ki-67^a^	Low (7)	189.20 ± 53.96	3.00 ± 0.00
	Moderate (15)	186.00 ± 45.53	4.20 ± 1.78
	High (78)	204.30 ± 61.46	3.90 ± 1.50
Chosen treatment	FAC^b^ (8)	206. 90 ± 75.83	3.80 ± 1.34
	AC-T^c^ (44)	183.80 ± 51.86	3.80 ± 1.29
Scheme	Only RT (48)	211.30 ± 63.42	4.40 ± 2.50

^a^Ki-67 = proliferation index: Ki-67 < 10% is low; Ki-67 of 10–25 is moderate; Ki-67 > 25 is high; ^b^FAC: fluorouracil, doxorubicin, and cyclophosphamide; ^c^AC-T: doxorubicin, cyclophosphamide, and taxol; RT: radiotherapy; QT: chemotherapy; *n*: number of individuals with the characteristic. ^∗^Significant at *P* < 0.05 in relation to progesterone positive receptor; ^∗∗∗^ *P* < 0.001 in relation to estrogen positive receptor; Mann Whitney *U* test was the test applied to evaluate the table's variables.

**Table 2:** DNA damage (mean ± SD) evaluation in peripheral blood of patients with breast cancer before, during, and after treatment and nonexposed control using comet assay.

Groups	Comet assay (100 cells/individual)	
	Damage index (0–400)	Damage frequency (%)
Control	22.90 ± 19.31	14.53 ± 8.24
*Patients*		
Before treatment^a^	180.90 ± 53.67^∗∗∗^	91.57 ± 13.94^∗∗∗^
Chemotherapy^b^	187.2 ± 56.61^∗∗∗^	94.83 ± 6.80^∗∗∗^
Before radiotherapy^c^	156.70 ± 69.68^∗∗∗^	73.10 ± 21.54^∗∗∗^
Radiotherapy^d^	189.60 ± 66.61^∗∗∗^	86.07 ± 12.34^∗∗∗^
After radiotherapy^e^	229.10 ± 47.93^∗∗∗^	90.20 ± 12.23^∗∗∗^

^a^Patient at the time of diagnosis; ^b^3 weeks after beginning chemotherapy; ^c^Before radiotherapy and after chemotherapy; ^d^3 weeks after beginning radiotherapy; ^e^21 days after the end of radiotherapy; ^∗∗∗^Significant at *P* < 0.001 in relation to control group (ANOVA and Kruskal-Wallis).

**Table 3:** DNA damage and cell death evaluated using micronucleus test in buccal in patients with breast cancer before, during, and after treatment and nonexposed control.

Parameters	Control	Patients				
		Before treatment^a^	Chemotherapy^b^	Before radiotherapy^c^	Radiotherapy^d^	After radiotherapy^e^
*DNA damage*						
Micronucleus	1.76 ± 1.30	3.93 ± 1.50^f^	4.00 ± 1.14^h^	5.53 ± 2.77^h^	7.60 ± 3.19^h, i^	8.16 ± 3.69^h, i^
Buds	2.43 ± 1.71	2.56 ± 1.59	1.90 ± 1.18	2.96 ± 2.55	4.06 ± 2.72	6.06 ± 3.37^h, i, and j^
Binucleated cells	5.33 ± 2.23	7.16 ± 4.99	9.93 ± 3.42^g^	9.10 ± 6.17	14.80 ± 16.75^g, i^	18.40 ± 17.03^h, i^
*Cell death*						
Condensed chromatin + karyorrhectic cells	195.50 ± 112.50	272.80 ± 105.10	412.50 ± 110.50^h, i^	340.80 ± 200.90	389.70 ± 228.8^g^	457.40 ± 276.00^h^
Pyknotic cells	1.70 ± 3.40	14.50 ± 5.50^h^	16.97 ± 4.99^h^	27.90 ± 35.20^g^	35.40 ± 37.50^h, i^	52.40 ± 52.3^h, i^
Karyolitic cells	53.80 ± 38.60	97.70 ± 63.80^f^	63.07 ± 23.30	121.50 ± 99.90^f^	163.50 ± 126.00^h^	226.80 ± 229.00^h^

Values represent the mean ± SD of 2000 buccal cells analyzed. ^a^Patient at the time of diagnosis; ^b^3 weeks after beginning chemotherapy; ^c^Before radiotherapy and after chemotherapy; ^d^3 weeks after beginning radiotherapy; ^e^21 days after the end of radiotherapy; ^f^Significant at *P* < 0.05; ^g^*P* < 0.01; ^h^*P* < 0.001 in relation to control group. ^i^Significant at *P* < 0.05 in relation to the group: before treatment. ^j^Significant at *P* < 0.05 in relation to the group: radiotherapy (ANOVA, Kruskal-Wallis).
